# Revisiting informed consent in forensic genomics in light of current technologies and the times

**DOI:** 10.1007/s00414-023-02947-w

**Published:** 2023-01-16

**Authors:** Bruce Budowle, Antti Sajantila

**Affiliations:** 1 Department of Forensic Medicine, University of Helsinki, Helsinki, Finland; 2 Forensic Medicine Unit, Finnish Institute for Health and Welfare, Helsinki, Finland

**Keywords:** Informed consent, Genetic information, Bioinformatics, Genetic genealogy, Risk, Benefit

## Abstract

Informed consent is based on basic ethical principles that should be considered when conducting biomedical and behavioral research involving human subjects. These principles—respect, beneficence, and justice—form the foundations of informed consent which in itself is grounded on three fundamental elements: information, comprehension, and voluntary participation. While informed consent has focused on human subjects and research, the practice has been adopted willingly in the forensic science arena primarily to acquire reference samples from family members to assist in identifying missing persons. With advances in molecular biology technologies, data mining, and access to metadata, it is important to assess whether the past informed consent process and in particular associated risks are concomitant with these increased capabilities. Given the state-of-the-art, areas in which informed consent may need to be modified and augmented are as follows: reference samples from family members in missing persons or unidentified human remains cases; targeted analysis of an individual(s) during forensic genetic genealogy cases to reduce an investigative burden; donors who provide their samples for validation studies (to include population studies and entry into databases that would be applied to forensic statistical calculations) to support implementation of procedures and operations of the forensic laboratory; family members that may contribute samples or obtain genetic information from a molecular autopsy; and use of medical and other acquired samples that could be informative for identification purposes. The informed consent process should cover (1) purpose for collection of samples; (2) process to analyze the samples (to include type of data); (3) benefits (to donor, target, family, community, etc. as applicable); (4) risks (to donor, target, family, community, etc. as applicable); (5) access to data/reports by the donor; (6) sample disposition; (7) removal of data process (i.e., expungement); (8) process to ask questions/assessment of comprehension; (9) follow-up processes; and (10) voluntary, signed, and dated consent. Issues surrounding these topics are discussed with an emphasis on addressing risk factors. Addressing informed consent will allow human subjects to make decisions voluntarily and with autonomy as well as secure the use of samples for intended use.

## Introduction

The predominately used markers for human identity testing are the autosomal short tandem repeat (aSTR) loci. These loci have relatively low to no positive predictive power to disclose personally identifiable information (PII) or personal data, such as health data or personal traits, about an individual. Personal data refer to “any information relating to an identified or identifiable natural person... if it can be directly or indirectly be used to identify a person or persons” [1]. The aSTRs can be (and are) used for kinship analyses (almost exclusively for first degree relative associations), and they do have a low predictive power for population affinity [2–4]. The same features of low to no positive predictive power regarding personal data can be attributed to Y-STRs. The Y-STRs can be more informative for inferring population affinity [5, 6] and extending the reach of kinship along the paternal line [7–11] than can be achieved with aSTRs. The maternal line counterpart, the mitochondrial DNA (mtDNA) genome, may provide some genetic information about the health and risk of an individual, although the positive predictive value still is low, and mechanisms for deleting known disease-associated variants have been proposed for forensic analyses [12, 13]. The value of mtDNA for estimation or prediction of population affinity and extended kinship is similar to that of Y-STRs [14–17]. One could take the position that these risks are nominal, are well managed in the forensic and medicolegal communities, and thus may or may not need to be disclosed when obtaining informed consent to acquire reference samples from individuals to perform applicable forensic casework.

### Principles and foundations of informed consent

Informed consent has its origins with the Nuremburg Code [18] (although the principles may have been developed earlier (see [19])) and has been codified by the Belmont Report [20], the Declaration of Helsinki [21, 22], many agencies and organizations (for example, see [23, 24]) and more recently the European Society of Human Genetics addressing direct to consumer (DTC) testing [25], to name a few. Additionally, these principles have been applied broadly to research on human subjects beyond medical research [1]. All espouse basic ethical principles that should be considered when conducting biomedical and behavioral research involving human subjects. These principles, outlined in the Belmont Report, are (1) respect for the individual to make adequately informed judgments freely (i.e., voluntarily and with autonomy) as well as to protect children and those persons with diminished capacity to effect autonomy; additionally worth noting is that as information/technology changes the individual (i.e., donor) may need to be updated to effect sound judgment decision processes; (2) beneficence particularly in the concept of do no harm, while maximizing potential benefits (taking note to avoid overestimation of benefits) and minimizing potential harm considering individual and society levels; and (3) justice so that all are treated fairly or equally.

These principles form the foundations of informed consent which in itself is grounded on three fundamental elements: information, comprehension, and voluntary participation. Informed consent procedures and supporting documents should provide sufficient information so participants can be made aware of the purpose and the research to be performed and the associated benefits and risks. The principle of transparency requires that information be conveyed in a comprehensible manner [26], and it falls on the researcher to ascertain that the potential participant understands the information. Risks should be well-described, and as risks become more severe, the requirement that they are comprehended should be rigorously followed. The agreement by the person to partake in the research study must be voluntarily given and free from coercion and undue influence. The participants should be provided ample opportunity to ask questions, seek to document the opportunity, and be allowed to withdraw at any time from the research. Foremost, personal data should be safeguarded to protect the integrity, dignity, and rights of individuals [1, 26]. Consent must be informed following the above principles for establishing a legal basis for processing samples and data [26].

While the principles and practices of informed consent were developed with medical and behavioral research in mind, they have been adopted willingly in the forensic science arena primarily to acquire reference samples from family members to assist in identifying missing persons (although some countries also obtain informed consent for reference samples from convicted offenders; see [27]). Thus, the forensic science community would appear to have embraced that the same principles of informed consent for medical research on humans also should be applied for practical work for human identification. Although the forensic community has been proactive with informed consent in missing persons and human remains cases, one could envision a sort of waiver of the consent requirement because (a) no foreseeable harm is expected to result from the sampling and analyses, and (b) sampling is permitted, e.g., by law or other regulations, or (c) if an ethical review committee has approved the non-disclosure of certain information [28].

There are examples of consent forms, frequently asked questions (FAQs) and information on processes, and/or recommended policies from a number of sources such as International Committee of the Red Cross (ICRC) [29], Interpol’s I-Familia [30, 31], Armed Forces DNA Identification Laboratory [32, 33], Budowle et al. [34], Australian Federal Police-National DNA Program for Unidentified and Missing Persons [35], and FBI’s Missing Persons Program [36] to name a few. Various aspects of informed consent for missing person identification have been summarized well by Katsanis et al. [37]. The information in these and other documents form a good basis to follow and need not be repeated herein. However, depending on the document(s), risk disclosure may be minimal or not stated, likely because the chance and/or severity of risk to an individual(s) were considered nominal at best. Additionally, as Katsanis et al. [37] point out “Unlike biomedical research requirements, the description of risks to the participant is not required by law.”

### Enhanced technical capabilities

With advances in molecular biology technologies, data mining, and access to metadata, it is important to assess whether the past informed consent process and in particular associated risks are concomitant with these increased capabilities. In more recent years, genetic markers (single nucleotide polymorphisms (SNPs)) have been used to predict externally visible traits, such as eye color, hair color, skin pigmentation, and freckles, to support investigative leads [38–43]. This technology, which is known as forensic DNA phenotyping [42], can be considered the forerunner to predict some personal information about an individual(s). Predicting these visible traits may not be considered a privacy risk as the features are readily identifiable without genetic testing, and an evidence sample has no rights [38]. Additionally, the use of such data can reduce bias by re-focusing investigations away from minority or vulnerable populations ([38], see [44] for more in-depth discussion on defining vulnerable populations), similar to how a Y-STR haplotype was used in the Vaastraa case to indicate that the donor of biological evidence was not from an immigrant population but from Western Europe [45]. However, some have asserted that forensic DNA phenotyping (visible features and particularly with biogeographical ancestry or ancestry informative markers [46, 47]) could contribute to the risk of stigmatization [48–51]. Beyond technical issues of, for example, causative versus non-causative SNPs, there are aspects that may be considered regarding the use, maintenance, and extended applications of such forensic predictive data that extend to the entire legal system to include “ethical principles such as autonomy, justice, dignity, confidentiality, and solidarity; legal principles such as due process and proportionality; and democratic values such as equality, transparency, and pluralism” as well as privacy [50]. Indeed, some of these aspects are integral to a proper informed consent and accentuate the need moving forward to enhance the informed consent process.

Forensic genomics capabilities have expanded to where it is now feasible to sequence an entire human genome by massively parallel sequencing, scan the genome with microarrays, and/or perform targeted sequencing of a relatively large number of loci or entire genes of reference and evidence samples [52–58]. Thus, vast amounts of genetic data can be generated at an individual or population level and potential access to such data may bring substantial benefits. However, access to the data (and samples) also pose greater risks than were not conceivable with STRs and mtDNA to those individuals who have been typed as well as under some certain circumstances the relatives of the genetically analyzed individuals. Consideration and disclosure of risks are important and should not be thought of an obstacle to dissuade participants but instead to ensure that they are well-informed to make autonomous decisions. Additionally, good stewardship can serve to protect the overall use of the technology for the intended benefits afforded.

It is not easy to predict future capabilities and associated efforts and thus previous informed consents should not be condemned for not being sufficiently informative about current advances. But all should be mindful of possible limitations of the use of legacy samples and data. Depending on the language in previous informed consents, it may be necessary to modify or expand documentation and/or processes in a continuous manner to address the enhanced genetic tools and risks of today [59]. Also, the donors of samples that were collected previously and archived may not be proper to use, given at least the spirit of informed consent. However, proportionality principles may tip the balance otherwise. Proportionality in this context could be that the actions proposed or taken should be weighed between the good that can be generated and the harm that may be caused. For example, it would be difficult for law enforcement to unlearn or ignore investigative leads, if the donor subsequently withdrew consent. It would be incumbent upon those who maintain the samples to reach back to update and likely obtain a new informed consent, if feasible or to consider the need to do so based on proportionality (a complex issue in itself for legacy samples and legacy data).

### Casework areas impacted by informed consent

There are at least four forensic scenarios in which informed consent may need to be modified and augmented based on these new capabilities: (1) reference samples from family members in missing persons or unidentified human remains cases; (2) targeted analysis of an individual(s) during forensic genetic genealogy (FGG) cases to reduce an investigative burden; (3) donors who provide their samples for validation studies (to include population studies and entry into databases that would be applied to forensic statistical calculations) to support implementation of procedures and operations of the forensic laboratory; and (4) family members that may contribute samples or obtain genetic information from a molecular autopsy [60]. While there are similar requirements for informed consent among these four scenarios, the latter (#4) likely should include a need for genetic counseling, informing relatives of potential harm (also applies to #1), and discussion about disposition and use of postmortem tissue samples that may be collected during and stored after autopsy [61].

While the purpose and benefits with advanced forensic genomic data may still be the same for these four areas as they have been since their inception, the informed consent clearly would have to be updated to describe the new technologies and how they are to be used to include the aims of use (or project), scope of use (or project), and type of data (see [26, 32]). The areas of informed consent that likely would require more thought are risks to the individual, risks to family members, perhaps risks to society, and particularly the issue that sample anonymity may be difficult to maintain and how readily easy it has become to mine genomic data and determine associated medical risks, biogeographical ancestry, kinship, etc. with these capabilities. While some risks may be considered low probability, but as conveyed in the Belmont Report [20], as the severity of the risk increases, the disclosure of the risk should be considered part of the information provided to the donors. One could consider disclosing reasonable risks versus low likelihood risks (possibly because the mechanisms in place by the laboratory system substantially reduce the chance of the particular risk from occurring) and disclose only those risks that are more likely to occur. However, with the potential of security compromises [62] and relative ease to mine data and determine source of an anonymized sample, a more exhaustive risk list with probability of occurrence (either qualitatively or quantitatively) may be more prudent to provide with informed consent. Katsanis et al. [37] advocate that it “is tantamount in bioethical principles for informed consent, allowing a potential participant to weigh the pros and cons of participation.” We concur and strongly urge those obtaining informed consent to put more effort into risk and benefit assessments to adequately inform sample donors.

### Informed consent process

The informed consent process should cover (1) purpose for collection of samples; (2) process to analyze the samples (to include type of data); (3) benefits (to donor, target, family, community, etc. as applicable); (4) risks (to donor, target, family, community, etc. as applicable); (5) access to data/reports by the donor; (6) sample disposition; (7) removal of data process (i.e., expungement); (8) process to ask questions/assessment of comprehension; (9) follow-up processes; and (10) voluntary, signed, and dated consent (Table 1). These topics need to be conveyed in lay terms to achieve comprehension.

**Table 1:** Major topics for consideration in the informed consent form and/or FAQs

Areas of informed consent	Considerations
Purpose	Missing person identification; assist in solving a crime; validation of a new methodology; contribute to research on improving sensitivity or detection from trace samples; allele frequency and population reference database; humanitarian purposes (missing persons, human trafficking, etc.); to determine genetic factors contributing to cause of death (or to suspected maltreatment), etc.
Technology/analytical process	Methodology used (e.g., MPS; Capillary Electrophoresis); type of data/genetic markers (SNPs; STRs; Biometric markers, etc.); algorithms or software tools to determine identity or kinship or genotype-phenotype etc.; databases that may be searched; limitations^#^, etc.
Benefits if contribute*	Potential to identify a missing relative; potential to identify perpetrator of a crime; potential to bring resolution to victims, families, and the community; potential for increased safety and security; to contribute to the research, development and implementation of enhanced forensic capabilities; identify genetic markers that pose health risk to individual or family members; mitigation plans that reduce risk; etc.**
Risks if contribute*	Discomfort during sampling; potential loss of sample; lack of anonymity; disclosure of data (by court order or inadvertently); Potential discrimination (e.g., unintended kinship, unintended biogeographical ancestry, vulnerable populations, personal health data, discovery of non-biological relationships with believed relatives); misuse by unauthorized individuals of sample or data; not properly informed about risk of data analyses beyond intended purpose (disclosed or obtained subsequently by donor); bad actors; maintaining sample or not disposing of sample in accordance with law or policy; maintaining data or not withdrawing data in accordance with law or policy; security breaches; data in private databases; data handled by private citizens; mitigation plans that reduce risk; etc.
Access to data	Used only for intended purpose; who may or will have access in laboratory or criminal justice system; donor provided data; donor not provided the data; research data that may be shared; confirmation or verification of informed consent; etc.
Sample disposition	Right to withdraw; maintained indefinitely and why; destroyed and at what time interval; destroyed upon case resolution; etc.
Removal of data process	Right to withdraw; expungement policy; withdrawal request policy; duration sample data may be maintained; etc.
Comprehension assessment	Frequently asked questions provided; mechanism for donor to ask questions; discussion with donor; access to genetic counselor; etc.
Follow up processes	Point of contact; only meet when sample is collected; no further contact; provide updates or report to donor; upon technology enhancement additional approval needed; etc.
Voluntary consent***	No coercion; freely obtained; signed consent form; trauma centric approaches; etc.

^#^An example of a limitation is standard technologies may not be able to distinguish by kinship analyses same sex siblings who perished together during a mass disaster. Other meta data or known direct reference samples would be needed potentially to resolve their identities

*Note the consequences of not contributing could be included in this table as they may be considered to contribute to coercion of the individual

**Benefits may not be directly to the donor, although identifying a missing relative or contributing to validation and implementation of a method donor can be envisioned as a benefit

***A possible risk of not providing a sample is that one’s relative may not be identified [30, 31]. However, just as is considered for collecting samples from certain subpopulations, one could suggest that families of missing persons (both in a disaster victim identification scenario and in a single individual situation) are a vulnerable group and thus there may be need to have special consideration to ensure that they are free of coercion [37]. At the time of a traumatic event, decision-making may be compromised, which could ensue for a long time period. Sample collectors should be trained with interpersonal skills in engaging donors that convey empathy, compassion, and clarity [63]. Additionally, economically disadvantaged or less educated or uneducated individuals may be considered part of a vulnerable group. The same could hold for a laboratory employee asked to donate a sample for validation purposes if, for example, his/her job/promotion might be interpreted to be affected by participation. Additionally, individuals approached for targeted sequencing for FGG may be considered a vulnerable population and proper informed consent is essential regarding coercion vs free choice.

Our goal is to raise awareness. The circumstances can be quite varied; but some existing consent forms and FAQs already form a good foundation that may only need to be updated or expanded accordingly. The information could be contained within a consent form. But given the degree of explanation of the purpose, the technology (and its limitations), benefits, risks, and accompanying processes placing such information in the consent form may become unwieldy. An accompanying FAQs document could contain most of the necessary information. However, since ensuring comprehension is an important aspect of proper informed consent, it may be moot about separating consent documentation and information or containing all in one document. We leave it to those involved in obtaining informed consent to determine which approach is more practical for their systems. Instead, Table 1 lists the above ten areas and some topics/concepts for consideration to expand on to be fit for purpose. The topics are not exhaustive but attempt to cover major topics.

Risk is likely higher with acquisition of more genetic data and thus important to consider when obtaining informed consent. The laboratory or whomever seeks consent should determine if occurrence of risk is low, moderate, or high and whether the impact would be low, moderate, or severe. Such assessments can help guide whether the risk should be disclosed or better yet identify measures to mitigate the risk. Below we discuss some areas of risk (not exhaustive) and in some cases what may be ways to mitigate the risks. While it is important to inform the participant about the potential risks, especially those risks that are highly likely and those that may have severe consequences, if they occur, the steps taken to mitigate risk could be perceived as a benefit.

### Privacy and security

The European Union’s General Data Protection Regulation (GDPR) [64], ICRC [26, 29], Budowle et al. [34], and I-Familia [30] stress that privacy by design and intended use are requisite for the use case. It is important to emphasize that the samples will be used for the intended purpose(s). Also, what may happen with the data obtained that are not requisite for the intended purpose should be disclosed by specific, blanket, broad information, tiered, presumed with explicit opt out opportunity, waived, or dynamic consent information [59] (see Table 2 for general privacy concepts). For example, it would be prudent to inform donors of reference samples for identifying relatives that their profiles may be searched in specific databases which may reside in another country, if that process is intended [30, 31, 34]. Another example would be during a disaster victim identification, where it may be determined that a father is not biologically related to one of his children. This finding is not directly relevant to the identification of the victim, and the donor(s) of the sample(s) should be made aware of the policy if such information will be or will not be disclosed (for example see [30]). We strongly advocate the latter to maintain the principle of intended use only, as well as privacy considerations.

**Table 2:** General data privacy concepts for best practices. Note that the concepts are not an exhaustive list but are provided for insight/examples into the processes

Concept	Description	Examples
Fit-for-purpose	Describe legitimate purposes and gather informed consent for collecting genomic and other personal data	Intended use, purpose of work, how it works, risks, benefits, follow-up if necessary or feasible
Data minimization	Collect only data necessary for the specified purpose	Intended use, measures to reduce risk of data and samples being accessed for unintended use; justifications for expansive sampling of data when more efficient means exist
Storage	Retain personal data and samples only as long as necessary for the specified purpose	Disposition, expungement, protocols for maintenance and disposal, designated appropriate personnel, security measures
Transparency	Describe lawful and/or ethical basis for processing personal or genomic data in a fair, clear and understandable manner	Openness, disclosure policies, accreditation, audits, proficiency testing
Confidentiality	Provide privacy and security of personal data and samples	Accreditation, audits, clearance levels for data and sample access, consequences for inappropriate disclosure, encryption, data that will and will not be disclosed
Accuracy	Ensure that personal data collected are correct, exact, up to date	Validation, known sources of error, processed files do not contain physical modifications or data perturbations, record keeping

Data security is critical for effective data protection [26]. Security measures, or more appropriately lack thereof, can be considered a serious risk, should be a major focus for handling of samples and data, and should be discussed when obtaining informed consent. Physical and IT measures and the degree that they are implemented impact risks (see Table 3 for general security measures). These measures may include training, access rights, physical security of the facility or data storage sites, password protection, encryption, data sharing agreements, destruction of personal data, data management, and retention procedures, to name a few [26]. De-identifying samples (and data) has been one of the primary ways to maintain sample anonymity. However, with technology today, the genetic data may be and likely will be sufficient to identify a donor [52]. Indeed, FGG is based on the use of genomic data to identify relatives of the sample donor and with public records triangulate to the donor [54]. Y-STRs alone have been used to do the same thing [11, 53]. Thus, the risk of identifying the donor (even if the genetic profile is anonymized in the traditional sense) is high. Of course, the risk could be reduced by not disclosing the genetic data or minimizing the data disclosed (part of a mitigation process).

**Table 3:** General data security measures to mitigate risk. Note that the measures are not an exhaustive list but are provided for insight/examples into the processes

Measure	Description or risk	Mitigation examples
Administrative controls	Data breach	Overall Information security program including training
		Policies and procedures to prevent, detect, contain and correct data violations
		Business/operation or data continuity plans and incident response plans
	Accountability	Designated database steward or risk officer
		Periodic external privacy impact studies
	Unauthorized access	Internal and external authorization processes
		Defined data retention policies for all files and stages of workflow
		Response plan for risk assessed or unperceived issues
Physical controls	Data access and availability	Designed database and storage mechanisms to limit proliferation of raw or processed data
		Inventory and logs on all systems that access and share data
		System, data and user level controls for system monitoring, encryption, tokenization, etc.
Technical controls	Unauthorized access	Authorization and authentication mechanisms such as multifactor sign in
		Registration of users
		System access logs
		Security of data when transmitted and during storage
		User initiated data deletion

There can be risks with public (see residual newborn bloodspot discussion below) and private sector groups who may exploit the samples and data for purposes not originally consented. If there are potential conflicts of interest with additional parties involved, those risks should be conveyed. Currently, most FGG genomic data are in the hands of the private sector (at least for now) but capabilities have been developed to transfer the methodology into the operational forensic laboratory [57, 65, 66]. National DNA databases have built secure measures to reduce unauthorized access, and, for example, there has been no report of a breach of CODIS. However, a private lab was able to gain access indirectly to CODIS profiles by convincing a government lab to search the National DNA Index System on its behalf [67]. Some of the private providers of FGG (either typing service or owners of databases) have continued to evolve to inform donors and users primarily about legal obligations, security, and privacy. For example, in its Terms of Service and Privacy Policy, GEDmatch.com [68] addresses the purpose of the database and its tools, and the acceptable samples for searching by law enforcement are from biological evidence from a violent crime or for identification of human remains. Users also are required to actively opt-in to sharing data for searching by law enforcement for violent crime investigations. Additionally, users agree they will not upload raw data in GEDmatch.com in violation of the intended use of the platform and that raw data will not be disclosed. Explicit guidance on risk minimization includes the following: the requirement to disclose data under legal obligations; privacy as a balance between risk of linking data to an individual and the need (i.e., purpose) to share information; conversion of raw data to a compressed binary format via a process called tokenization (which is not encryption in the usual sense but is an attempt to limit the ability to reverse engineer an originally uploaded file); deletion and archiving of data; the risk of hacking and decoding of data; access of data by GEDmatch personnel and volunteers on a need to know basis; use of data for internal research; the data will not be shared with third parties for research; and notification requirement if data are shared with third parties. Privately maintained data likely will vary substantially regarding security measures to protect data, and private entities should embrace informed consent and describe the use of acquired data for intended purposes.

### Anonymity or de-identification

Given sufficient genetic data, computational power, and accessible metadata, maintaining anonymity may no longer be achievable. Indeed, FGG and familial searching [69] demonstrate that a person can be identified with current tools. Thus, it may be necessary to move from the concept of anonymity to that of pseudonymization. Pseudonymization is defined as “the Processing of Personal Data in such a manner that the Personal Data can no longer be attributed to a specific Data Subject without the use of additional information, provided that such additional information is kept separately and is subject to technical and organizational measures to ensure that the Personal Data are not attributed to an identified or identifiable natural person” [26]. The European Union’s GDPR has endorsed pseudonymization as a measure to protect personal data (Article 4(5), Under Article 4(5) [64]). The capability to anonymize or better yet pseudonymize personal data should be conveyed to the human subject.

Measures to preserve privacy of genomic data are more complicated and require cryptographic techniques for data encryption and protection. Homomorphic encryption is one example of a mechanism to enable data analyses for forensic purposes and maintain pseudonymization [70]. Audits should be undertaken on a regular basis of holders of genomic data for forensic purposes to confirm that the data are secure, data are expunged, and the samples are used for only intended purposes according to policies or contracts in place. It also is important to disclose if audits are performed and relevant findings will or will not be disclosed. At a minimum, genomic data searched in private databases should be used for the intended purpose only and should be expunged when the search is complete (after an adequate time frame). Additionally, all new developments or capabilities should be evaluated via a Privacy Impact Assessment (PIA), or equivalent, to ensure that new feature(s) being included in the software accounts for privacy by design. PIA assessments also evaluate whether new capabilities may unintentionally introduce security vulnerabilities into the software. Regular database penetration testing strategies as part of a robust information security management system can help highlight security risks as well options for mitigation. Interestingly, how security risks are addressed could be perceived as a benefit (at least compared to other options for donor participation) if the measures taken provide more confidence of protection of personal data.

### Disclosure and disposition policies

Disclosure and mechanisms to enable or restrict disclosure of genetic data are critical issues. For example, one of us (BB) when becoming Director of the Center for Human Identification became aware that DNA profiles were placed in reports. The original intent may have been to be informative and helpful but may not have considered privacy issues and statutes (which can impact donors such as victims as well as the laboratory). As stated above, STRs (and mtDNA to a slightly greater extent) might have been considered low risk from a privacy perspective. But under the Federal DNA Identification Act 34 U.S.C. § 12592 and Texas Government Code § 411.153, unauthorized disclosure is prohibited and is a state jail felony. The practice of placing DNA results in reports was stopped. Presumably, similar legislation exists in other jurisdictions. Some jurisdictions still may include DNA profiles in reports and may be legally permissible to do so. However, even if not stated in a report, the data are still maintained in case files. One possible way to protect the privacy of individuals is to encrypt the DNA profiles in a manner that the genetic data are not disclosed but in a format that still allows for direct or relationship comparisons. A judge could order disclosure of the genetic data. Possibly a protective order could be placed on the genetic data; but once disclosed, the data are not under control of the laboratory, and thus there is a risk that the data are no longer secure, may be shared with others, may be used for purposes other than it was intended, etc. Disclosure policies and risks should be described to explain who may have access, under what general conditions, and in what data format. Additionally, the fact that disclosure of data can occur regardless of the laboratory’s efforts should be disclosed to the potential participant.

Disposition and particularly retention of the sample(s) and/or profile(s) can be considered a risk. Risk attributes with raw data, especially dense SNP data, are much higher than with STR markers. As long as samples and/or data are maintained, someone unauthorized or even an authorized person may gain access to the samples and/or data and use them beyond intended purposes. Duration of maintenance of the samples and/or data should be described. Policy and/or legislation may dictate the status of samples and profiles. One could see the value of maintaining the DNA sample ad infinitum for re-testing with new technologies or confirmation purposes as well as having as complete a genetic data set per individual for population analyses. Alternatively, there is potential risk that maintaining a sample may allow access for unintended or unauthorized purposes. So, disposal and expungement policies may be desirable. For example, there would not seem to be a need to maintain reference samples and profiles once an identification of human remains is made. One could consider maintaining the reference profiles if the remains were partial. But if an identification was made, then the profile from the remains could serve as a direct comparison profile for additional remains that may be recovered. This approach assumes that the identification is made with a high degree of confidence.

### Third-party usage

Another challenge to the protection of personal data (and foremost in maintaining the integrity and dignity of individuals) is that the data generated, for example, for a population study to establish allelic frequency data, may be submitted to third party databases to support forensic applications. The two most noted examples are the Y-STR Haplotype Reference Database (YHRD) [71] and the European DNA Profiling Group’s Mitochondrial DNA Population Database (EMPOP) [72] databases. These databases (and others, for example see [73, 74]) are invaluable resources that support the forensic DNA community. However, the good intentions of the operators of these databases and others can be marred if those in the supply chains of data do not obtain proper informed consent or do not consider in their informed consent (or by permissible legislation) first, the vulnerability of individuals or certain subpopulation groups such as Australia’s Aboriginal and Torres Strait Islander people, Native Americans, Saami in northern Europe, and Roma (see, for example, [75]). For databases such as EMPOP and YHRD that collect data based on population affinity, such vulnerable groups need to be represented, if possible, to perform statistical calculations that likely will be overstated for such groups if not adequately represented. As described in a Report of the International Bioethics Committee of UNESCO [44], “Vulnerability is an inescapable dimension of the life of individuals and the shaping of human relationships. To take into account human vulnerability acknowledges that we all may lack at some point the ability or the means to protect ourselves, our health and our well-being.” Additionally, “While some groups of people can always be considered vulnerable because of their status (e.g. children), others may be vulnerable in one situation but not in another. Therefore, vulnerability cannot be considered as a one-off concept.” Thus, it is incumbent upon scientists to assess vulnerability in each study undertaken. Criteria to consider for assessing vulnerability include the following: rights of every human being; marginalization of peoples; social and educational conditions; gender discrimination; personal liberty; hierarchical relationships; war; exploitation; to name a few [44]. A second consideration is the potential use of the data by a third party (i.e., the databases and the forensic community that rely on the database data). Both of these issues affect autonomy and especially once the personal data are shared. While the operators of the databases have a responsibility to ensure that proper informed consent has been obtained and already take measures to protect the data once in their possession, those scientists undertaking the population genetic studies can effect a better process by having proper and effective informed consent. Recognizing what constitutes a vulnerable population and how to convey in a comprehensible manner the intent, goals, processes, risks, and benefits, as well as engagement with the individuals, can go a long way to empower individuals with the autonomy to make decisions whether to contribute or not to a study without coercion. Regarding obtaining family reference samples from outside the country to identify human remains found within the country, Budowle et al. [43] (see appendix 5 FAQs) require that informed consent cover that the donor understands and agrees that the sample may be searched in national and non-national databases. It should be noted that population data that are published are intended to be used further than for just the sake of publication. In a similar vein, it may be inferred or those who perform population studies believe it to be inferred that data will be placed in certain third party databases and since publication of population data is conditioned on submission to respective databases, going forward, perhaps this third party aspect should be expressed explicitly. This recommendation is particularly critical with dense SNP data where anonymity may no longer be maintained. Lastly, while researchers collecting samples for forensic genetic analyses are obviously the main persons responsible for collecting informed consent and providing the necessary information in an unbiased and non-coercive way, journals publishing the data also can serve as gatekeepers. Most journals have policies that address informed consent predominately relying on the researcher to affirm conditions have been met. Further review of the policies may be worth considering given genomic advances and the blurred line between legally allowed and vulnerable populations.

### Considerations in medicolegal autopsy

A medicolegal autopsy may require collection of reference samples for human identification purposes, and the same informed consent approaches described above should apply. However, a molecular autopsy to assist in determining cause and/or manner of death, predominately in unexplained or sudden unexpected deaths, relies on genetic diagnostics [76–78]. In this medicolegal area, genetic markers with positive predictive power are employed and that information may impact (i.e., be a risk or reduce harm) family members of the deceased. Family members, a priori, would have increased risk based on sharing of genomes. Also, some family members may be requested to provide a reference sample(s) to better interpret the potential risk of observed genetic variants. Sajantila and Budowle [60] described the risks about information disclosure as the medicolegal context (requests performed under judicial mandate or request) is different than for standard clinical work.

The risks include “(1) genetic information of deceased individuals resides in files of judicial institutions; (2) there is no institutional body within the judicial infrastructure who would naturally meet the medical/genetic consultation needs of the relatives, even if the relatives were to seek advice; and (3) the genetic information in the reports can be elicited to the public during court proceedings.” The risks to privacy are greater (especially with the ease of genetic data analyses and that a molecular autopsy would point directly to a gene(s) and specific genetic variants) and should be included in the informed consent documentation with a greater need by medicolegal personnel to ensure the information is comprehended by sample donors and potentially impacted family members. Additionally, although not part of informed consent, these genetic data may be disclosed publicly and/or reported to family members. There is little or no guidance, i.e., genetic counseling, to such individuals regarding the risks associated with the genetic findings.

This scenario of potential risk to family members gained during a molecular autopsy confounds the personal data integrity process, and the principle of proportionality applies here. We do not attempt to address the issue herein but intend to raise awareness of the complexities of genetic data obtained in the medicolegal context. As stated by Fellmann et al. [61], the focus of a medicolegal investigation is to ascertain whether the cause of death can be assigned to an underlying disease or if there are any legal implications, i.e., determining if the cause of death is natural as opposed to unnatural. Informing family members of the underlying cause of death is not necessarily part of the aim of the medicolegal investigation. These authors further point out that if the cause of death may be attributed to a genetic condition, there now is knowledge that relatives may be at increased risk and that knowledge could be particularly relevant if there is a treatment that may prevent family members from succumbing. Therefore, an autopsy may go beyond determining cause of death, and practitioners should consider informing family members that they may have an increased risk of developing the genetic disease (especially if that risk may be high). Informed consent is usually not required for postmortem investigations. It is not possible to obtain consent from a deceased individual and yet some relatives may have a vested interest in being informed about the genetic results obtained from the deceased individual. Fellmann et al. [61] advocate that if serious harm might be prevented through providing this knowledge to family members, proportionality would support disclosure as opposed to obligations to maintain confidentiality. We concur. However, access to postmortem tissue samples, who will be informed, and when and what information will be given are challenging issues. It will take much more thought than merely raising herein awareness of the complexities of informed consent and subsequent data sharing in the molecular autopsy context.

### Providing samples for validation studies

Often, laboratory personnel donate their DNA to support validation studies that are necessary for a quality system. One could submit that in countries where disclosure of such data is possible or expected to meet legal obligations and/or maintain transparency, there could be risk of access to the donors’ genetic data. It may seem that under such legal systems, consent is inferred as anyone employed in those forensic laboratories should be aware to some degree of disclosure requirements. However, it may be prudent to obtain informed consent from donors to validation studies as they may not fully appreciate the ramifications of disclosure and subsequent risks which are much greater with for example dense SNP data, as well as to ensure that agreement is not considered coercion. An alternate source of samples for research and validation studies has been from casework. Nonprobative samples often have been used for validation studies, for example samples from victims. If not already obtained, it may be prudent, going forward, to obtain informed consent to use such samples. In contrast, some jurisdictions may interpret use of these samples obtained without informed consent is legally and/or ethically permissible (see [79] as an example of use of vulnerable population samples without informed consent, but legally permissible, for research and validation studies).

### Risk from other sources

The ICRC [29] recommends disclosing data to donors who request the information, which is similar to policies of the DTC companies. With access to data, accuracy of results can be verified or challenged. There is merit in this approach as it could garner more participation and greater transparency. Alternatively, this approach could be considered undue influence prompting people to participate without fully considering the associated risks. The data are complex, and as such there may be a tendency to consider access to the data to be a low risk because of the belief that a bioinformatician is required to interpret the genomic data. A number of students today, however, are being trained on bioinformatics and thus the pool of experts is increasing and likely will continue to increase. Moreover, genomic data mining is feasible by untrained individuals. There are online tools that allow individuals independently to upload their genomic data and determine genetic variants that pose health risks, determine bioancestry, and find relatives (e.g., [80–82]). These tools can be quite empowering and, in some cases, quite helpful (e.g., finding an adopted person’s biological parents, although in some cases the parent(s) may not want to be located). However, the untrained may (and likely will) misinterpret the findings which can lead to false positives, false negatives, and over- or underestimation of the genetic risk [82]. Importantly, the emergence of off-the-shelf tools and automation workflows likely will reduce the need for bioinformatics or even genetic training. If genomic data are shared with the participant, proper cautions and disclaimers by the laboratory should be in the informed consent. Of course, sharing the data which then are subsequently used by the donor for another purpose could be interpreted as facilitating use for an unintended purpose (balanced against the autonomy of the donor). Since the genomic data are intended for forensic purposes only, it may be more prudent to not provide to the donor the raw data or profile. Access or no access to data and accordingly the associated risks should be part of informed consent.

There is little discussion about the risk of bad actors, likely because most of us believe the risk to be low and predicting such activity is difficult. After all, ultimately, trust and integrity are essential to the profession, and violation of these principles is considered reprehensible. However, there have been fraudulent or corrupt individuals, most of which are single individuals (e.g., see [83]), although not always by a single actor (e.g., see [84]). Although such occurrences are unlikely, the consequences often are quite severe. Completely eliminating the risk of a single bad actor may not be feasible. Security risks and measures to reduce bad actions can be instituted and possibly described during the informed consent process.

### Risk assessment

In order to determine risk and possible severity, a risk analysis should be part of the laboratory, criminal justice system, and/or agencies processes. It is important to convey risks and consequences to properly inform donors. While it is not the purpose of this paper to instruct on how to perform a risk assessment, it is important to stress herein that risk be assessed to address the likelihood of an event and the consequences that may occur [26]. For risk assessment, one should consider (1) the risk and under what circumstances the risk may occur; (2) the consequences should the risk occur; (3) the likelihood of the consequences to occur; and (4) what processes or mechanisms are in place or need to be put in place to control or minimize the risk [85]. These assessments are part of a good quality assurance program to address root cause analysis, corrective action, and preventive action [86]. One approach is a probability/consequence matrix method to assess the overall severity of the contributing factors to each potential risk [87].

### Access to samples collected for other intended purposes

Throughout this paper, informed consent has been discussed in light of applications that forensic geneticists and medicolegal scientists may have more direct interactions. Perhaps there is a fifth scenario to consider in which another sample resource that may be used for law enforcement purposes that may require discussion on informed consent. That resource is the vast medical and clinical samples maintained in the healthcare arena. As an example, many countries require newborn screening, within the first 48 h, for a number of medical and genetic disorders [88, 89]. These analyses are legally mandated, and there seems to be little controversy (and possibly little awareness) regarding sample collection and the analyses. However, the residual blood spots (RBS), also known as Guthrie cards, have been retained for years creating a substantial biological resource that may be exploited for other purposes. Indeed, these samples have been used successfully, for example, for disaster victim identification [90, 91]. Very recently, the New Jersey State Police subpoenaed the State’s Newborn Screening Laboratory to obtain a specific blood card to hone in on the donor’s father during an FGG investigation [92, 93]. The lead appears to have been successful; however, a lawsuit has been filed claiming that law enforcement violated its constitutional obligation of demonstrating probable cause to obtain a warrant, as well as there was no informed consent regarding the use of the samples [93]. In a similar fashion over a decade ago in the case of Beleno v. Texas Department of State Health Services (No. 5:09-cv-00188-FB (W.D.Tex., San Antonio Division filed Mar. 12, 2009)), five families filed a lawsuit claiming that the State Agency and Texas A&M University violated federal and state law by using the legally mandated newborn blood spots for unspecified research without the parents’ knowledge or consent [94]. Apparently, the samples were provided to various agencies for research, one being AFDIL to generate a population database [88, 94]. The issue of informed consent was side-stepped in the case, but one consequence was that between 4 and 5 million blood cards that existed at the time were destroyed. The storage and use of RBS samples for secondary purposes raise socio-ethical and public policy issues around privacy, transparency, and autonomy. In the absence of clear guidelines around the use of RBS, common positions held by the research community are (a) RBS samples are considered community property since everyone benefits from the research, and (b) anonymizing the de-identified data is sufficient to protect the privacy of the donor. However, with genomic capabilities today, anonymization may no longer be feasible. In cases where RBS samples are used for the specific forensic purpose of identification, one could envision requiring informed consent from the parents (on behalf of the child) as well as the ability to “opt-in” to forensic use. We are not weighing in on whether these samples can be used for purposes beyond the intended purpose, whether use is legally permissible (although note that such samples have been used legally under exigent circumstances, such as a disaster (see [90])), or whether informed consent is required for newborn screening (all of which shall be left to other discussions); we instead are raising awareness that such samples exist (to include many other medical samples such as biopsy samples or samples collected for whole genome sequencing studies [95] and for that matter the non-medical collection of samples by the military) and may be sought to support criminal and humanitarian investigations. Laboratories should seek guidance on the usage of such samples and be aware that they may not have been obtained with informed consent before undertaking analyses as the consequences could be severe. We strongly advocate that the principle of intended use is maintained, and proactively looking forward with the option to actively opt-in to secondary use of a sample in order to preserve privacy.

## Conclusion

Our goal is to raise awareness and facilitate discussion on these important and fundamental aspects of personal data, the role of informed consent, and the need to update the process to be concomitant with current technological capabilities and the times. “The development of new technologies allowing for easier and faster Processing of ever-increasing quantities of Personal Data in an inter-connected world has given rise to concerns about the possible intrusion into the private sphere of individuals” [26]. We are not suggesting that every issue raised herein must be addressed in every informed consent. Instead, we urge that forensic scientists and their law enforcement/legal system counterparts consider these various issues to ensure that a proper informed consent is met relevant to the intended work. While an in-depth informed consent may be perceived as cumbersome, the benefits in the short and long term are individuals will be protected and allowed to make sound decisions (i.e., autonomy) and the use of powerful technologies to investigate crime and identify missing persons and human remains will be sustained. There have been forays recently with using an electronic informed consent (i.e., e-consent), for example, in clinical research and in biobanks [96, 97]. An e-consent makes use of digital technologies incorporating multimedia to convey information and obtain informed consent. There are some indications that e-consent could enhance the informed consent process, particularly for format, subject understanding through greater interactivity, storage, and potential streamlining. While there are some issues to address, either generally or on a case-by-case basis, such as security, legal validity, acceptability by ethics boards, computer literacy with some groups, trust, privacy, and cost, to name a few [98], some guidance for developing and enacting an effective e-consent has been provided [99]. The forensic community should consider investigating e-consent as it may facilitate the task of fully meeting informed consent.

As with all use of genetic data, balance is an important aspect. Discussed herein are examples in which samples may be used legally even though informed consent may not have been obtained and a scenario in which law enforcement may have obtained information and the donor may choose to withdraw consent after the fact. Thus, there may be situations where informed consent, at least in spirit or by intent, may not be necessary or overridden for the greater good. Within the European Economic Area, the entering into force of the GDPR saw a distinct move away from the privileged status of informed consent as the basis for processing personal data. This action does not, however, mean that informed consent should not be used when genetic data are collected from the donor. To the contrary, it is a prerequisite for ethical use of the data as stated, for example, in the WMA Declaration of Taipei version [100]. In terms of the GDPR, it is possible to process genetic data based on law which provides for the purpose and further conditions of the use and eventual possibilities for storing the data (Art. 6.1. (e) or 6.1. (c) together with Art. 9.2. (g)). Genetic data still may be processed on the basis of consent (6.1. (a) and 9.2 (a)), which has to conform to the requirements of the GDPR (Art. 7). The GDPR also opens the door for a broad consent presupposing that it keeps within recognized ethical standards of scientific research (Recital 33). A dynamic electronic consent, in particular, is recommended by the European Data Protection Board.

Vulnerability should be considered and addressed to obtain a valid consent. The social, cultural, and religious norms of the potential vulnerable group of which the human subject(s) belongs should be considered, and these individuals should be treated with dignity and respect. While individualized informed consent is desired, there can be circumstances that may make it difficult to meet this goal. For example, in a mass disaster where there are exigent circumstances and a chaotic environment, it may be more effective to inform the public and/or those directly impacted individuals through media so that they may have an exigent opportunity to understand and agree to intended use(s), processes, risks and benefits of the sample, subsequent generated data, and other personal data that may be gathered. All activities need to be weighed under the principle of proportionality [26]. Finally, we all must hold ourselves accountable to the principles of informed consent and actions or consequences that may ensue with the samples and data obtained. If we do so, we can protect the individuals, give respect and dignity to the individuals, and ultimately contribute effectively to our mission of applying forensic science to support criminal and humanitarian investigations.

## References

[1] Finnish National Board on Research Integrity TENK guidelines 2019, The ethical principles of research with human participants and ethical review in the human sciences in Finland. At: https://tenk.fi/sites/default/files/202101/Ethical_review_in_human_sciences_2020.pdf; accessed last 24/07/2022

[2] Klintschar M, Füredi S, Egyed B, Reichenpfader B, Kleiber M (2003). Estimating the ethnic origin (EEO) of individuals using short tandem repeat loci of forensic relevance. Int Congress Series.

[3] Fosella X, Marroni F, Manzoni S, Verzeletti A, De Ferrai F, Cerri N, Presciuttini S (2004). Assigning individuals to ethnic groups based on 13 STR loci. Int Congress Series.

[4] Lowe A, Urquhart A, Foreman LA (2001). Evett IW (2011) Inferring ethnic origin by means of an STR profile. Forensic Sci Int.

[5] Jobling MA, Tyler-Smith C (2003). The human Y chromosome: an evolutionary marker comes of age. Nat Rev Genet.

[6] Purps J, Siegert S, Willuweit S, Nagy M, Alves C, Salazar R, Angustia SMT, Santos LH (2014). A global analysis of Y-chromosomal haplotype diversity for 23 STR loci. Forens Sci Int Genet.

[7] Chakraborty R (1985). Paternity testing with genetic markers: are Y-linked genes more efficient than autosomal ones?. Am J Med Genet.

[8] Foster EA, Jobling MA, Taylor PG, Donnelly P, de Knijff P, Mieremet R, Zerjal T, Tyler-Smith C (1998). Jefferson fathered slave’s last child. Nature.

[9] Kayser M, Roewer L, Hedman M, Henke L, Henke J, Brauer S, Krüger C, Krawczak M, Nagy M, Dobosz T, Szibor R, de Knijff P, Stoneking M, Sajantila A (2000). Characteristics and frequency of germline mutations at microsatellite loci from the human Y chromosome, as revealed by direct observation in father/son pairs. Am J Hum Genet.

[10] Liu H, Li X, Mulero J, Carbonaro A, Short M, Ge J (2016). A convenient guideline to determine if two Y-STR profiles are from the same lineage. Electrophoresis.

[11] Ge J, Budowle B (2021). Forensic investigation approaches of searching relatives in DNA databases. J Forens Sci.

[12] Budowle B, Gyllensten U, Chakraborty R, Allen M (2005). Forensic analysis of the mitochondrial coding region and association to disease. Int J Leg Med.

[13] Marshall C, Sturk-Andreaggi K, Ring JD, Dür A, Parson W (2020). Pathogenic variant filtering for mitochondrial genome haplotype reporting. Genes.

[14] Sajantila A, Lahermo P, Anttinen T, Lukka M, Sistonen P, Savontaus ML, Aula P, Beckman L, Tranebjaerg L, Gedde-Dahl T, Issel-Tarver L, DiRienzo A, Pääbo S (1995). Genes and languages in Europe: an analysis of mitochondrial lineages. Genome Res.

[15] Delfin F, Myles S, Choi Y, Hughes D, Illek R, van Oven M, Pakendorf B, Kayser M, Stoneking M (2012). Bridging near and remote Oceania: mtDNA and NRY variation in the Solomon Islands. Mol Biol Evol.

[16] Dulik MC, Zhadanov SI, Osipova LP, Askapuli A, Gau L, Gokcumen O, Rubinstein S, Schurr TG (2012). Mitochondrial DNA and Y chromosome variation provides evidence for a recent common ancestry between Native Americans and Indigenous Altaians. Am J Hum Genet.

[17] Översti S, Onkamo P, Stoljarova M, Budowle B, Sajantila A, Palo J (2017). Local demographic patterns buried in the present mtDNA genome pool: Finland as an example. Sci. Rep..

[18] Nuremberg Code. At: https://encyclopedia.ushmm.org/content/en/article/the-nuremberg-code; accessed last 24/07/2022

[19] Ghooi RB (2011). The Nuremberg Code–a critique. Perspectives in Clinical Research.

[20] The Belmont Report: Ethical Principles and Guidelines for the Protection of Human Subjects of Research. The National Commission for the Protection of Human Subjects of Biomedical and Behavioral Research. DHEW Publication No. (OS) 78-0012. U.S. Government Printing Office, 30 (1978). Washington. DC. CHECK FOR APRIL.

[21] https://www.wma.net/policies-post/wma-declaration-of-helsinki-ethical-principles-for-medical-research-involving-human-subjects/; accessed last 24/07/2022.

[22] World Medical Association (2013). Declaration of Helsinki: ethical principles for medical research involving human subjects. JAMA.

[23] Health and Human Services, Informed Consent FAQs. At: https://www.hhs.gov/ohrp/regulations-and-policy/guidance/faq/informed-consent/index.html; accessed last 24/07/2022.

[24] National Institutes of Health, Help me understand genetics genetic testing, U.S. National Library of Medicine, MedlinePlus Genetics. At: https://medlineplus.gov/genetics/; accessed last 24/07/2022.

[25] European Society of Human Genetics (2010). Statement of the ESHG on direct-to-consumer genetic testing for health-related purposes. European Journal of Human Genetics.

[26] Handbook on data protection in humanitarian action, Second Edition, Brussels Privacy Hub, the Vrije Universiteit Brussel (Free University of Brussels or VUB) and the Data Protection Office of the International Committee of the Red Cross (ICRC), Geneva, Switzerland, Kuner C, Marelli M, editors, At: https://www.icrc.org/en/data-protection-humanitarian-action-handbook; accessed last 24/07/2022.

[27] Best Practice for Forensic DNA Databases 2017, A report by the Forensic Genetics Policy Initiative. At: http://dnapolicyinitiative.org/wp-content/uploads/2017/08/BestPractice-Report-plus-cover-final.pdf; accessed last 24/07/2022.

[28] International ethical guidelines for biomedical research involving human subjects. Council of International Organisations of Medical Sciences (CIOMS) in collaboration with the World Health Organization (WHO) Geneva, Switzerland (2002) At: https://cioms.ch/wp-content/uploads/2016/08/International_Ethical_Guidelines_for_Biomedical_Research_Involving_Human_Subjects.pdf; accessed last 24/07/2022.

[29] International Committee of the Red Cross (ICRC) Missing people, DNA analysis and identification of human remains, A guide to best practice in armed conflicts and other situations of armed violence Second edition 2009; Available at: http:// www.cmu.edu/chrs/conferences/eppi/docs/ICRC-DNA-Analysis.pdf; accessed last 24/07/2022.

[30] Interpol I-Familia, At: https://www.interpol.int/en/How-we-work/Forensics/I-Familia; accessed last 24/07/2022.

[31] Laurent F-X, Fischer A, Oldt RF, Kanthaswamy S, Buckleton JS, Hitchin S (2022). Streamlining the decision-making process for international DNA kinship matching using Worldwide allele frequencies and tailored cutoff log10LR thresholds. Forens Sci Int Genet.

[32] Armed Forces Medical Examiner System, DNA Identification Laboratory Fact Sheet (2018) FAQs. At: https://www.dpaa.mil/Resources/Fact-Sheets/Article-View/Article/590581/armed-forces-medical-examiner-system-dna-identification-laboratory/; accessed last 24/07/2022.

[33] Family Reference Collection Form, Armed Forces Medical Examiner System, DNA Identification Laboratory. At: file:///C:/Users/Owner/Downloads/2016%20Donor%20Consent%20Form%20V90%20DNA%20Form%20332%20June%202016%20(1).pdf; accessed last 24/07/2022.

[34] Budowle B, Buś MM, Josserand MA, Peters DL (2020). A standalone humanitarian DNA identification database system to increase identification of human remains of foreign nationals. Int J Leg Med.

[35] Australian Federal Police - National DNA Program for Unidentified and Missing Persons. At: https://www.missingpersons.gov.au/support/national-dna-program-unidentified-and-missing-persons#faq-DNA-testing-information; accessed last 24/07/2022.

[36] FBI – National Missing Persons Program. At: https://www.fbi.gov/services/laboratory/biometric-analysis/codis/codis-and-ndis-fact-sheet#National-Missing%20Person%20Program; accessed last 24/07/2022.

[37] Katsanis SH, Snyder L, Arnholt K, Mundorf AZ (2018). Consent process for US-based family reference DNA samples. Forens Sci Int Genet.

[38] Kayser M, Schneider PM (2009). DNA-based prediction of human externally visible characteristics in forensics: motivations, scientific challenges, and ethical considerations. Forens Sci Int Genet.

[39] Walsh S, Liu F, Ballantyne KN, van Oven M, Lao O, Kayser M (2011). IrisPlex: a sensitive DNA tool for accurate prediction of blue and brown eye colour in the absence of ancestry information. Forensic Sci Int Genet.

[40] Walsh S, Liu F, Wollstein A, Kovatsi L, Ralf A, Kosiniak-Kamysz A (2013). The HIrisPlex system for simultaneous prediction of hair and eye colour from DNA. Forens Sci Int Genet.

[41] Xiong Z, Gao X, Chen Y, Feng Z, Pan S (2022). Combining genome-wide association studies highlight novel loci involved in human facial variation. Nat Comm.

[42] Kayser M (2015). Forensic DNA phenotyping: predicting human appearance from crime scene material for investigative purposes. Forensic Sci Int Genet.

[43] Chaitanya L, Breslin K, Zuñiga S, Wirken L, Pośpiech E, Kukla-Bartoszek M, Sijen T, Knijff P, Liu F, Branicki W, Kayser M, Walsh S (2018). The HIrisPlex-S system for eye, hair and skin colour prediction from DNA: introduction and forensic developmental validation. Forensic Sci Int Genet.

[44] The principle of respect for Human vulnerability and personal integrity. Report of the International Bioethics Committee of UNESCO (IBC) (2013). At: https://unesdoc.unesco.org/ark:/48223/pf0000219494); last accessed 17/08/22.

[45] Kayser M (2017). Forensic use of Y-chromosome DNA: a general overview. Hum Genet.

[46] Pakstis AJ, Speed WC, Soundararajan U, Rajeevan H, Kidd JR, Li H, Kidd KK (2019). Population relationships based on 170 ancestry snps from the combined kidd and seldin panels. Sci Rep.

[47] de la Puente M, Ruiz-Ramírez J, Ambroa-Conde A, Xavier C, Pardo-Seco J, Álvarez-Dios J (2021). Development and evaluation of the ancestry informative marker panel of the VISAGE basic tool. Genes.

[48] M’charek A, Toom V, Prainsack B (2012). Bracketing off population does not advance ethical reflection on EVCs: A reply to Kayser and Schneider. Forens Sci Int Genet.

[49] Schneider PM, Prainsack B, Kayser M (2019). The use of forensic DNA phenotyping in predicting appearance and biogeographic ancestry. Dtsch Arztebl Int.

[50] Toom V, Wienroth M, M'charek A, Prainsack B, Williams R, Duster T, Heinemann T, Kruse C, Machado H, Murphy E (2016). Approaching ethical, legal and social issues of emerging forensic DNA phenotyping (FDP) technologies comprehensively: reply to 'Forensic DNA phenotyping: Predicting human appearance from crime scene material for investigative purposes' by Manfred Kayser. Forens Sci Int Genet.

[51] Tozzo P, Politi C, Delicati A, Gabbin A, Caenazzo L (2021). External visible characteristics prediction through SNPs analysis in the forensic setting: a review. Frontiers in Bioscience-Landmark.

[52] Erlich Y, Shor T, Pe’er I, Carmi S (2018). Identity inference of genomic data using long-range familial searches. Science.

[53] Fitzpatrick C (2018) Solving the Phoenix Canal Murders. ISHI News. At: https://www.ishinews.com/solving-the-phoenix-canal-murders/; accessed last 24/07/2022.

[54] Greytak EM, Moore C, Armentrout SL (2019). Genetic genealogy for cold case and active investigations. Forens Sci Int.

[55] Kling D, Phillips C, Kennett D, Tillmar A (2021). Investigative genetic genealogy: current methods, knowledge and practice. Forens Sci Int Genet.

[56] Tillmar A, Fagerholm SA, Staaf J, Sjölund P, Ansell R (2021). Getting the conclusive lead with investigative genetic genealogy – a successful case study of a 16 year old double murder in Sweden. Forens Sci Int Genet.

[57] Tillmar A, Sturk-Andreaggi K, Daniels-Higginbotham J, Thomas JT (1968) Marshall C (2021) The FORCE Panel: an all-in-one SNP marker set for confirming investigative genetic genealogy leads and for general forensic applications. Genes 12(12) 10.3390/genes12121968 PMC8702142 34946917

[58] Dowdeswell TL (2022). Forensic genetic genealogy: a profile of cases solved. Forens Sci Int Genet.

[59] Human genetics and genomics in South Africa: ethical, legal and social implications – consensus study (2018) Department of Science and Technology Republic of South Africa, Academy of Science of South Africa; At: https://research.assaf.org.za/handle/20.500.11911/106; last accessed 17/08/22.

[60] Sajantila A, Budowle B (2016). Postmortem medico-legal genetic diagnostics also require reporting guidance. Eur J Hum Genet.

[61] Fellmann F, van El CG, Charron P, Michaud K, Howard HC, Boers SN, Clarke AJ (2019). European recommendations integrating genetic testing into multidisciplinary management of sudden cardiac death. Eur J Hum Genet.

[62] Edge MD, Coop G (2020). Attacks on genetic privacy via uploads to genealogical databases. eLife.

[63] Wayland S, Ward J (2022). Dreading yet hoping: traumatic loss impacted by reference DNA sample collection for families of missing people. Front Psychiatry.

[64] European Union’s General Data Protection Regulations At: https://gdpr-info.eu/art-4-gdpr/; accessed last 24/07/2022.

[65] Gorden EM, Greytak EM, Sturk-Andreaggi K, Cady J, McMahon TP, Armentrout S, Marshall C (2022). Extended kinship analysis of historical remains using SNP capture. Forens Sci Int Genet.

[66] Snedecor J, Fennell T, Stadick S, Homer N, Antunes J, Stephens K, Holt C (2022). Fast and accurate kinship estimation using sparse SNPs in relatively large database searches. Forens Sci Int Genet (submitted)..

[67] Lydell Grant case Lydell Grant case. At: https://www.law.umich.edu/special/exoneration/Pages/casedetail.aspx?caseid=5980; accessed last 24/07/2022.

[68] GEDmatch.com Terms of Service and Privacy Policy. At: https://www.gedmatch.com/terms-of-service-privacy-policy; accessed last 24/07/2022

[69] Familial DNA Searching: Current Approaches, Final Report. National Institute of Justice, Office of Investigative and Forensic Sciences, 2015; At: https://rti.connectsolutions.com/p49iz1rzbpi/; accessed last 24/07/2022

[70] Kim M, Lauter K (2015). Private genome analysis through homomorphic encryption. BMC Medical Informatics and Decision Making.

[71] Willuweit S, Roewer L, International Forensic Y Chromosome User Group (2007). Y chromosome haplotype reference database (YHRD): update. Forens Sci Int Genet.

[72] Parson W, Dur A (2007). EMPOP—A forensic mtDNA database. Forens Sci Int Genet.

[73] Bodner M, Bastisch I, Butler JM, Fimmers R, Gill P, Gusmão L, Morling N, Phillips C, Prinz M, Schneider PM, Parson W (2016). Recommendations of the DNA Commission of the International Society for Forensic Genetics (ISFG) on quality control of autosomal Short Tandem Repeat allele frequency databasing (STRidER). Forens Sci Int Gen.

[74] Strider 2.0. at: https://strider.online/; accessed last 24/07/2022

[75] Lipphardt V, Surdu M, Ellebrecht N, Pfaffelhuber P, Wienroth M, Rappold GA (2021). Europe's Roma people are vulnerable to poor practice in genetics. Nature.

[76] Ackerman MJ, Tester DJ, Porter CJ, Edwards WD (1999). Molecular diagnosis of the inherited long-QT syndrome in a woman who died after near-drowning. N Engl J Med.

[77] Ackerman MJ, Siu BL, Sturner WQ (2001). Postmortem molecular analysis of SCN5A defects in sudden infant death syndrome. JAMA.

[78] Koren G, Cairns J, Chitayat D, Gaedigk A, Leeder SJ (2006). Pharmacogenetics of morphine poisoning in a breastfed neonate of a codeine-prescribed mother. Lancet.

[79] Hopkins C, Taylor D, Hill K, Henry J (2019). Analysis of the South Australian Aboriginal population using the Global AIMs Nano ancestry test Forens Sci Int Genet.

[80] Bianchi L, Lio P (2007). Forensic DNA and bioinformatics. Brief Bioinform.

[81] Folkersen L, Pain O, Ingason A, Werge T, Lewis CM, Austin J (2020). Impute.me: an open-source, non-profit tool for using data from direct-to-consumer genetic testing to calculate and interpret polygenic risk scores. Front. Genet..

[82] Direct-to-consumer genomic testing, National Human Genome Research Institute, NIH. At: https://www.genome.gov/dna-day/15-ways/direct-to-consumer-genomic-testing; accessed last 24/07/2022.

[83] Griebel KK (2012) Fred Zain, the CSI effect, and a philosophical idea of justice: using West Virginia as a model for change. 114 W. Va. L. Rev. At: https://researchrepository.wvu.edu/wvlr/vol114/iss3/13; accessed last 24/07/2022.

[84] Budowle B, Carroll J, Weller T (2021) Final Report of Review and Audit of Selected Casework of the Firearms Examination Unit of the Forensic Science Laboratory Division, Department of Forensic Sciences, District of Columbia, March 18, 2021. At: https://wtop.com/wp-content/uploads/2021/03/rondell_mcleod_dfs_court_filing_march_22.pdfBioinformatician needed to analyze data ref; accessed last 24/07/2022.

[85] Gonzalez Z (2022) Risk analysis: a comprehensive guide. At: https://safetyculture.com/topics/risk-analysis/; accessed last 24/07/2022.

[86] ISO 17025. At: https://www.iso.org/ISO-IEC-17025-testing-and-calibration-laboratories.html https://www.indeed.com/career-advice/career-development/risk-analysis-methods; accessed last 24/07/2022.

[87] Indeed Editorial Team (2021) 5 risk analysis methods and how to use them. At: https://www.indeed.com/career-advice/career-development/risk-analysis-methods; accessed last 24/07/2022.

[88] Cunningham S, O’Doherty KC, Sénécal K, Secko D, Avard D (2015). Public concerns regarding the storage and secondary uses of residual newborn bloodspots: an analysis of print media, legal cases, and public engagement activities. J Community Genet.

[89] Ngan O, Li CK (2020). Ethical issues of dried blood spot storage and its secondary use after newborn screening programme in Hong Kong. HK J Paediatr.

[90] Hartman D, Benton L, Morenos L, Beyer J, Spiden M, Stock A (2011). The importance of Guthrie cards and other medical samples for direct matching of disaster victims using DNA profiling. Forensic Sci Int.

[91] Monelius K, Lindblom B (2012). DNA analysis in disaster victim identification. Forensic Sci Med Pathol.

[92] Office of the Atlantic County Prosecutor. Brian Avis indicted on 8 counts for sexual assault of 10 year-old Brigantine girl & 5 year-old Galloway girl. At: https://www.acpo.org/brian-avis-indicted-on-8-counts-for-sexual-assaults-of-10-year-old-brigantine-girl-5-year-old-galloway-girl/; accessed last 02/08/2022.

[93] NJ police used baby DNA to investigate crimes, lawsuit claims. At: https://www.theverge.com/2022/7/29/23283837/nj-police-baby-dna-crimes-lawsuit-public-defender; accessed last 02/08/2022.

[94] Carnahan SJ (2011). Biobanking newborn bloodspots for genetic research without consent. J Health Care Law and Policy.

[95] Burkhi T (2022). UK explores whole-genome sequencing for newborn babies. Lancet.

[96] Gesualdo F, Daverio M, Palazzani L, Dimitriou D, Diez-Domingo J, Fons-Martinez J, Jackson S, Vignally P, Rizzo C, Tozzi AE (2021). Digital tools in the informed consent process: a systematic review. BMC Med Ethics.

[97] Guarino J, Parvanova I, Finkelstein J (2021) Characteristics of electronic informed consent platforms for consenting patients to research studies: a scoping review. In: Otero P et al (eds) MEDINFO 2021: One World, One Health – Global Partnership for Digital Innovation, 2022 International Medical Informatics Association (IMIA) and IOS Press, vol 290, pp 777–781 At: https://ebooks.iospress.nl/doi/10.3233/SHTI220184 10.3233/SHTI220184 35673123

[98] De Sutter E, Zaçe D, Boccia S, Di Pietro ML, Geerts D, Borry P, Huys I (2020). Implementation of electronic informed consent in biomedical research and stakeholders’ perspectives: systematic review. J Med Internet Res.

[99] US Food and Drug Administration (FDA) (2016) Use of electronic informed consent: questions and answers. Guidance for institutional review boards, investigators, and sponsors. At: https://www.fda.gov/regulatory-information/search-fda-guidance-documents/use-electronic-informed-consent-clinical-investigations-questions-and-answers. Accessed last 24/07/2022

[100] WMA Declaration of Taipei on ethical considerations regarding health databases and biobanks (2016); At: https://www.wma.net/policies-post/wma-declaration-of-taipei-on-ethical-considerations-regarding-health-databases-and-biobanks/; accessed last 09/08/22. 10.3917/jib.283.0113 29561093

